# A Systematic Review of Nudge Interventions to Optimize Medication Prescribing 

**DOI:** 10.3389/fphar.2022.798916

**Published:** 2022-01-25

**Authors:** Usman Talat, Kelly Ann Schmidtke, Saval Khanal, Amy Chan, Alice Turner, Robert Horne, Tim Chadborn, Natalie Gold, Anna Sallis, Ivo Vlaev

**Affiliations:** ^1^ Alliance Manchester Business School, The University of Manchester, Manchester, United Kingdom; ^2^ Warwick Medical School, Coventry, United Kingdom; ^3^ Warwick Business School, Coventry, United Kingdom; ^4^ School of Pharmacy, University of Auckland, Auckland, New Zealand; ^5^ Institute for Applied Health Research, University of Birmingham, Birmingham, United Kingdom; ^6^ UCL School of Pharmacy, University College London, London, United Kingdom; ^7^ Public Health England, London, United Kingdom; ^8^ London School of Economics and Political Science, Public Health England, London, United Kingdom; ^9^ Kantar Public, London, United Kingdom

**Keywords:** medical decision-making, systematic reviews, prescribing/use/costs, behavioural science, nudge

## Abstract

**Background:** The benefits of medication optimization are largely uncontroversial but difficult to achieve. Behavior change interventions aiming to optimize prescriber medication-related decisions, which do not forbid any option and that do not significantly change financial incentives, offer a promising way forward. These interventions are often referred to as nudges.

**Objective:** The current systematic literature review characterizes published studies describing nudge interventions to optimize medication prescribing by the behavioral determinants they intend to influence and the techniques they apply.

**Methods:** Four databases were searched (MEDLINE, Embase, PsychINFO, and CINAHL) to identify studies with nudge-type interventions aiming to optimize prescribing decisions. To describe the behavioral determinants that interventionists aimed to influence, data were extracted according to the Theoretical Domains Framework (TDF). To describe intervention techniques applied, data were extracted according to the Behavior Change Techniques (BCT) Taxonomy version 1 and MINDSPACE. Next, the recommended TDF-BCT mappings were used to appraise whether each intervention applied a sufficient array of techniques to influence all identified behavioral determinants.

**Results:** The current review located 15 studies comprised of 20 interventions. Of the 20 interventions, 16 interventions (80%) were effective. The behavior change techniques most often applied involved prompts (*n* = 13). The MINDSPACE contextual influencer most often applied involved defaults (*n* = 10). According to the recommended TDF-BCT mappings, only two interventions applied a sufficient array of behavior change techniques to address the behavioral determinants the interventionists aimed to influence.

**Conclusion:** The fact that so many interventions successfully changed prescriber behavior encourages the development of future behavior change interventions to optimize prescribing without mandates or financial incentives. The current review encourages interventionists to understand the behavioral determinants they are trying to affect, before the selection and application of techniques to change prescribing behaviors.

**Systematic Review Registration**: [https://www.crd.york.ac.uk/prospero/], identifier [CRD42020168006].

## 1 Introduction

To prescribe medications optimally healthcare practitioners must make decisions about whether prescribing medication is appropriate, and, if so, how much, how often, and what type of medications they use (National Institute for He, 2015). Behavior change interventions can optimize these decisions in terms of patient safety and cost-effectiveness (Michie et al., 2011; Faria et al., 2014). Behavior change interventions that do not forbid any options and do not significantly change economic incentives may be referred to as nudges (Thaler and Sunstein, 2009; Vlaev et al., 2016). The current systematic review is the first to synthesize information about behavior change interventions designed to optimize prescribing decisions, without forbidding options or changing economic incentives, according to theoretically and empirically informed frameworks that can inform future interventions.

The benefits of medication optimization are largely uncontroversial but challenging to achieve (Faria et al., 2014). This difficulty arises, in part, due to the many stakeholders (e.g., patients and clinicians) and processes involved (e.g., prescribing, dispensing, administering, monitoring, and record-keeping involved). To simplify this picture, the current review focuses on practitioner prescribing decisions. Other reviews have focused on a more specific set of intervention modes (e.g., e-prescribing) (Nuckols et al., 2014; Williams et al., 2020) or types of patients (e.g., patients with dementia) (McGrattan et al., 2017; ShafieeHanjani et al., 2019). They do not focus on interventions aiming to change prescribing behavior without forbidding options or changing economic incentives. Nudge interventions are appealing in bringing about better practice; where nudge interventions are effective, they are more likely to be socially and professionally acceptable than mandates (Nagtegaal et al., 2019).

Of course, whether an intervention succeeds depends on more than whether prescribers find it acceptable. A successful intervention must address existing reasons (also called barriers and facilitators) for whether a desirable behavior occurs (Hardeman et al., 2005). The Behavior Change Wheel (Michie et al., 2011) provides an empirically and theoretically supported guide that helps interventionists to identify these reasons and then to select techniques best suited to overcome them. For example, a prescriber may not know what medications the current guidelines recommend (a barrier related to knowledge), which could be overcome by making concise guidelines more accessible (Allen and Harkins, 2005; Elwyn et al., 2016). Alternatively, e.g., a prescriber may not feel comfortable recommending a more optimal medication because their patients seem uncomfortable considering new options (a barrier related to social influences), which could be overcome by encouraging shared decision making (Stead et al., 2017).

As more than one reason may underlie suboptimal prescribing, many interventions are complex in the sense that they involve one or more techniques to overcome each identified reason (Craig et al., 2008; Michie et al., 2009). Addressing all the reasons for suboptimal prescribing simultaneously is important, as any unaddressed reason can prevent change. For example, prescribers likely need both sufficient knowledge regarding what medications to prescribe and sufficient social support to do so.

The current review has two objectives. The main objective is to describe the techniques applied in nudge-type behavior change interventions to optimize practitioners’ prescribing and whether those interventions were effective. The second objective is to describe whether the previous interventions applied a sufficient array of techniques to address all of their identified determinants (Elwyn et al., 2016).

## 2 Materials and Methods

The current review is reported according to the Preferred Reporting Items for Systematic Reviews and Meta-Analyses guidelines (Moher et al., 2009). The review was registered on the February 27, 2020 on the International Prospective Register of Systematic Reviews (ID: CRD42020168006).

### 2.1 Eligibility

The inclusion and exclusion criteria were defined according to the PICOS framework, see Supplementary Appendix 1. We included studies reporting on evaluated behavior change interventions to optimize medication prescribing for people with a legal ability to prescribe, e.g., clinicians. Additionally, studies needed to describe potential behavioral determinants of suboptimal prescribing that interventionists intended to influence. The interventions were restricted to those that did not forbid choices and did not significantly change economic incentives. Both randomized and non-randomized studies were included, so long as there was a comparison condition, e.g., non-randomised studies could include before/after comparisons. The search was restricted to studies published in peer-reviewed journals and written in the English language, as no translation services were available to the research team. The initial publication year was bound by each database’s restrictions.

### 2.2 Information Sources and Search Strategy

The information sources and search strategies were designed in collaboration with a university librarian to include all PICOS characteristics combined using Boolean variables. The final search was conducted by one author (SK) on the 5th of August 2019 over the following databases: MEDLINE (1946 to June 2019), Embase (1980 to June 2019), PsycINFO (1806 to June 2019), and CINAHL (1961 to June 2019). An example of the search conducted in Medline (Ovid) is provided in Supplementary Appendix 1. SK exported the retrieved study titles and abstracts into Endnote (version X9.2 for Windows & Mac, released June 11, 2019) and deleted duplicates.

### 2.3 Study Selection

Two reviewers (UT and SK) assessed the eligibility of articles. First, they independently screened the titles and abstracts according to the inclusion/exclusion criteria. Their initial agreement is described using a percentage (number of agreements divided by the total number of decisions). Disagreements were resolved *via* consensus-based discussions with IV acting as an arbitrator. The reasons for removing titles and abstracts were not recorded. The remaining full-texts were reviewed by UT, SK, IV, and KS. Final decisions were made *via* consensus-based discussions. The reasons for removing full-text articles were recorded.

### 2.4 Data Collection Process and Data Items

**FIGURE 1:**
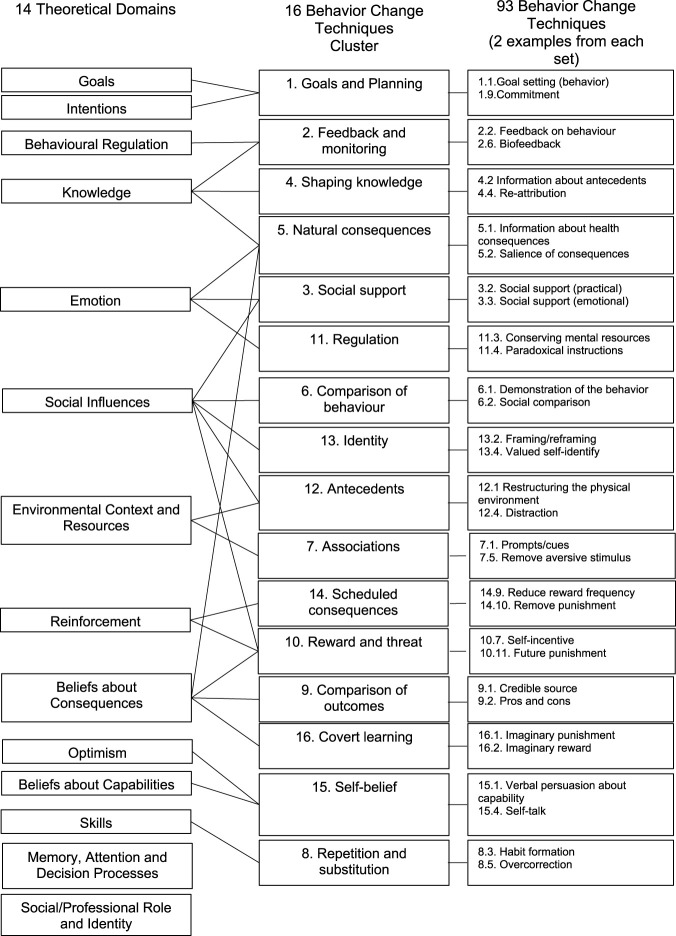
Recommended connections between the Behavior Change Techniques version 1, Technique Clusters, and Theoretical Domains Framework Domains.

Three data extraction templates were designed to meet our objectives. The first was used to compile the information about the behavioral determinants each intervention aimed to influence from each study’s introduction. As recommended in the Behaviour Change Wheel’s manual, the possible determinants are described using the Theoretical Domains Framework (TDF) (Cane et al., 2012). The TDF is a cross-disciplinary synthesis of 112 unique constructs of behavior change into 14 theoretically informed domains, also called determinants, e.g., “knowledge” and “social Influences.” All 14 determinants are listed at the left of Figure 1.

The second data extraction template compiled information about the behavior change techniques applied using the Behavior Change Techniques (BCTs) Taxonomy version 1 (Michie et al., 2013). This taxonomy includes 93 empirically supported techniques that on their own have the potential to change behavior. To increase the usability of the BCTs Taxonomy, the 93 techniques have been hierarchically arranged into a more manageable set of 16 clusters (Cane et al., 2015). The techniques are typically referred to using the same verbiage, along with a number that identifies their cluster and number within that cluster. For example, the “2.3 self-monitoring of behavior” technique is the third technique listed in the second cluster (the Feedback and Monitoring cluster), and the “3.2 social support (practical)” technique is the second technique listed in the third cluster (the Social Support cluster). The 16 clusters and examples of techniques within them are provided in the middle and right of Figure 1.

The relationships between the theoretical domains and the techniques best suited to influence them are indicated in the connecting lines between the first and second columns in Figure 1. For instance, the “knowledge” determinant is linked to techniques in the Feedback and Monitoring cluster. A potential problem arises here, as no techniques are linked to the following two determinants: “memory, attention, and decision processes” and “social/professional role and identity.” Thus, there is a need for another framework to fill this gap.

To fill this gap, the third data extraction template complied information about concepts described in the behavioral economics literature. While these concepts are not explicitly called techniques, some can certainly be used to change behavior (Vlaev et al., 2016; Tagliabue et al., 2019). For instance, the MINDSPACE framework summarizes nine of the most robust and non-coercive influences on behavior (Dolan et al., 2010; Kahneman, 2011; Dolan et al., 2012). Each letter in MINDSPACE stands for a different contextual influencer including *messenger, incentives, norms, defaults, salience, priming, affect, commitments,* and *ego*, see Table 1. While some contextual influencers may already be included as BCTs in the taxonomy, others are not. For example, the *commitment* contextual influencer is likely captured by the “1.9 commitment” technique, but it is difficult to say which techniques capture *defaults* and *priming*. Coding the techniques and contextual influencers in this review allows us to describe those that have been applied in previous interventions, and to highlight those that are potentially underutilized and unevaluated.

**TABLE 1:** MINDSPACE contextual influencers.

Contextual Influencers	Definition informed by Dolan 2010 (Dolan et al., 2010)
Messenger	We are heavily influenced by who communicates information. We are affected by the perceived authority of the messenger (whether formal or informal). Demographic and behavioral similarities between the expert and the recipient can improve the effectiveness of the intervention
Incentive	Our evaluations of outcomes and responses to incentives are shaped by mental biases such as strongly avoiding losses, overweighting small probabilities (hence why lotteries may act as a powerful motivation), mental accounting (we tend to allocate different rewards/payoffs to discrete mental accounts), and present bias (we prefer more immediate rewards)
Norms	We are strongly influenced by what others do. People often take their understanding of social norms from the behavior of others. Relate the norm to your target audience as much as possible and consider social networks
Defaults	We “go with the flow” of pre-set options. Many decisions we take every day have a default option, whether we recognize it or not. Defaults are the options that are pre-selected if an individual does not make an active choice
Salience	Our attention is drawn to what is novel and seems relevant to us. Our behavior is greatly influenced by what our attention is drawn to. People are more likely to register stimuli that are novel, accessible, simple, and relevant
Priming	Our acts are often sub-consciously influenced by cues that activate (prime) concepts in our memory. Priming often occurs when external, situational cues activate a goal, which affects information processing and behavior to achieve the primed goal representation. People’s subsequent behavior may be altered if they are first exposed to certain sights, words or sensations
Affect	Our emotional associations can powerfully shape our actions. Emotional responses to words, images and events can be very rapid and automatic
Commitment	We seek to be consistent with our public promises, and to reciprocate acts. People use commitment devices to achieve long-term goals. One common commitment device is to make commitments public, since breaking the commitment will lead to significant reputational damage. Creating an action-plan which specifies who needs to do what, when and where is also a commitment device. A final aspect of commitment is our strong instinct for reciprocity, which is linked to a desire for fairness
Ego	We act in ways that make us feel better about ourselves. We tend to behave in a way that supports the impression of a positive and consistent self-image

In summary, there were 14 decisions made about the theoretical domains identified in each study (i.e., yes or no for each domain), 93 about the behavior change techniques applied in each intervention, and nine about the contextual influencers applied in each intervention. UT and KAS extracted these data independently. Their initial agreements are described using percentages (number of agreements divided by the total number of decisions). Disagreements were resolved *via* consensus-based discussions.

Data were also extracted to describe broader study characteristics (publication year, country, setting, study design, and study duration) and to describe whether each intervention optimized prescriber behavior. The effectiveness of the intervention was determined by each study’s authors in terms of their given comparison condition; for single-arm studies, the comparisons were between the pre-intervention measures and the post-intervention measures. The study designs were classified using the Scottish Intercollegiate Guidelines Network’s (SIGN’s) study design algorithm (Miller, 2002). The quality of evidence generated by each study was appraised using SIGN’s critical appraisal checklists and notes. A brief description of each intervention was compiled according to the Template for Intervention Description and Replication (TIDieR) checklist (Hoffmann et al., 2014).

### 2.5 Synthesis of Results

Narrative syntheses and tallies are used to summarize the behavioral determinants identified in each study, the techniques and contextual influencers applied in each intervention, and whether each intervention was effective. We also examined whether each intervention applied a sufficient array of recommended techniques to address its identified determinants. A meta-analysis was not conducted due to the heterogeneous nature of the study methods and outcomes.

## 3 Results

### 3.1 Study Selection

**FIGURE 2:**
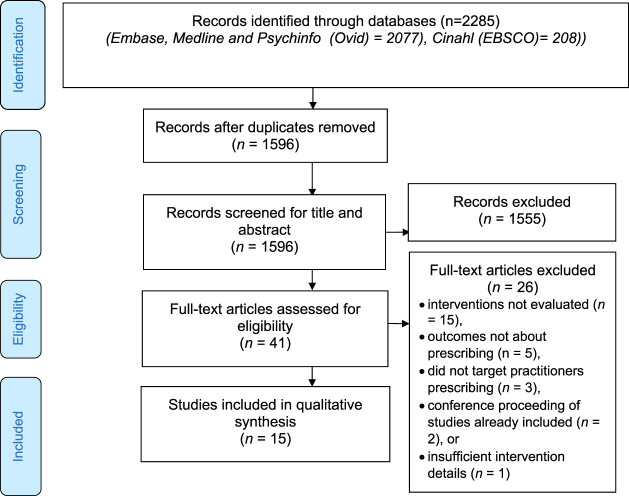
PRISMA flowchart.

The study selection process is summarized in Figure 2. The initial search identified 1,596 articles with duplicates removed. Of the 1,596 articles, 1,555 were removed after reviewing the titles/abstracts. The initial agreement on the titles and abstracts was high (99%). A further 26 articles were removed after reviewing the full texts. A list of these articles and the reasons for their exclusions are provided in Supplementary Appendix 2. Ultimately, 15 articles that evaluated interventions to optimize prescribing were included (Bourdeaux et al., 2014; Harewood et al., 2011; Isenberg et al., 2018; Lemiengre et al., 2018; Mafi et al., 2018; Malhotra et al., 2016; Meeker et al., 2014; Musgrove et al., 2018; O'Connor et al., 2009; Patel et al., 2018; Patel et al., 2017; Presseau et al., 2018; Sacarny et al., 2018; Shakespeare et al., 2019; Yadav et al., 2019).

### 3.2 Study Characteristics

Study characteristics are described in Table 2. The studies were published between 2009 and 2018, inclusive. Most of them were conducted in North America (*n* = 10), followed by Europe (*n* = 4), and Australia (*n* = 1). The median duration of the studies was 12 months (range = 2–67). More studies were conducted in secondary care (*n* = 8) than in primary care (*n* = 5). While 11 studies evaluated a single intervention, four evaluated more, and the total number of evaluated interventions was 20. The interventions are described in greater depth later in the Section 3. Of the 20 interventions described, 16 (80%) were effective. Complete descriptions of each intervention according to the TIDieR checklist are provided in Supplementary Appendix 3 *.*

**TABLE 2:** Study characteristics.

Study	Country	Study duration (Months)	Setting	Study design^a^
Bourdeaux et al. (2014)	United Kingdom	67	Secondary Care	ITS
Harewood et al. (2011)	Ireland	2	Secondary Care	ITS
Isenberg et al. (2018)	United States	36	Tertiary Care	ITS
Lemiengre et al. (2018)	Belgium	12	Primary Care	C-RCT
Mafi et al. (2018)	United States	20	Secondary Care	NRT
Malhotra et al. (2016)	United States	24	Secondary Care	ITS
Meeker et al. (2014)	United States	12	Primary Care	I-RCT
Musgrove et al. (2018)	United States	12	Secondary Care	ITS
O'Connor et al. (2009)	Canada	50	Secondary Care	ITS
Patel et al. (2018)	United States	2	Primary Care	C-RCT
Patel et al. (2017)	United States	31	Primary Care	NRT
Presseau et al. (2018)	United Kingdom	12	Primary Care	C-RCT
Sacarney et al. (2018)	United States	9	Primary Care and Secondary Care	I-RCT
Shakespeare et al. (2019)	Australia	12	Secondary Care	ITS
Yadav et al. (2019)	United States	20	Secondary Care	C-RCT

a C-RCT, Cluster-Randomized Controlled Trial, I-RCT, Individually—Randomized Controlled trial; ITS, interrupted time Series, and NRT, Non-Randomized Trial.

### 3.3 Quality Assessment

Eight of the 15 studies used designs that could be evaluated with one of the SIGN’s checklists, see Table 2. Six of these studies were rated as generating high-quality evidence, one as acceptable, and one as low. The high-quality studies generally consisted of RCT methods carried out with adequate concealment. The low-quality study lacked reporting for many elements, e.g., for whether the only difference between groups is the treatment under investigation, and for the percentage of participants who dropped out before the study was completed. The remaining seven studies used an interrupted time-series design, for which SIGN does not make a checklist available and considers as entailing a higher risk of bias. The full quality assessments are provided in Supplementary Appendix 4.

### 3.4 Behavioral Determinants

Table 3 summarizes the data extracted to meet the review objectives. The behavioral determinants identified for each study are described in the second column. The reviewers’ initial agreements were high (84%). While all studies posited some reasons for suboptimal prescribing, none included detailed information about how they identified the determinants. Only one study, Presseau et al., included references to previous publications that did explain how the determinants of suboptimal prescribing were identified, specifically regarding prescribing medications to treat diabetes (Presseau et al., 2018).

**TABLE 3:** Behavioral determinants identified in each study, along with the techniques recommended to influence them, the behavior change techniques/*contextual influencers* applied in each intervention, whether the techniques applied addressed all the determinants identified, and whether the interventions were effective.

Study	Behavioral determinants identified	Behavior change techniques^a^ applied	*Contextual influencers applied*	Did the behavior change techniques applied address all determinants?^b^	Was the intervention effective?
Bourdeaux et al. (2014)	-Memory attention and decision processes	Intervention 1: 7.1 Prompts/cues	*Defaults*	Not possible	Yes
		Intervention 2: 7.3 Reduce prompts/cues	*Defaults*	Not possible	Yes
Harewood et al. (2011)	-Environmental context and resources	12.1 Restructuring the physical environment	*Defaults and Priming*	Not possible	Yes
	-Memory attention and decision processes				
Isenberg et al. (2018)	-Environmental context and resources	2.2 Feedback on behavior	*Messenger, Defaults, and Salience*	Completely	Yes
	-Knowledge	4.1 Instruction on how to perform behavior			
		7.1 Prompts/cues			
		12. Adding objects			
Lemiengre et al. (2018)	-Emotions	Intervention 1:	*Affect*	Partially	No
	-Environmental context and resources	4.1 Instruction on how to perform behavior			
	-Knowledge	4.2 Information about antecedents			
	-Social influences	12.5 Adding objects			
		Intervention 2: 3.2 Social support (practical)	*Affect*	Partially	No
		3.3 Social support (emotional)			
		12.5 Adding objects			
		Intervention 3: 3.2 Social support (practical)	*Affect*	Completely	No
		3.3 Social support (emotional)			
		4.1 Instruction on how to perform behavior			
		4.2 Information about antecedents			
		12.5 Adding objects			
Mafi et al. (2018)	-Knowledge	1.9 Commitment	*Messenger, Norms and Commitments*	Not possible	Yes
	-Memory attention and decision processes	2.2 Feedback on behavior			
		4.1 Instruction on how to perform behavior			
		7.1 Prompts/cues			
Malhotra et al. (2016)	-Memory attention and decision processes	7.1 Prompts/cues	*Defaults and Salience*	Not possible	Yes
	-Social influences				
Meeker et al. (2014)	-Goals	1.9 Commitment	*Norms, Salience and Commitments*	Not possible	Yes
	-Memory attention and decision processes	2.1 Monitoring of behavior, without feedback			
	-Social influences	3.2 Social support (practical)			
	-Social/Professional Role and Identity	3.3 Social support (emotional)			
		5.1 Information about health consequences			
		6.3 Information about others’ approval			
Musgrove et al. (2018)	-Knowledge	4. Information about antecedents	*Salience*	Not possible	Yes
	-Memory attention and decision processes	7.1 Prompts/cues			
O’Connor (2009)	-Memory attention and decision processes	7.1 Prompts/cues	*Defaults*	Not possible	YES
Patel et al. (2018)	-Environmental context and resources	Intervention 1: 4.2 Information about antecedents	*Norms and Defaults*	Not possible	Yes
	-Memory attention and decision processes	6.3 Information about others’ approval			
		7.1 Prompts/cues			
		12.5 Adding objects			
		Intervention 2:	*Norms and Defaults*	Not possible	Yes
		2.2 Feedback on behavior			
		4.2 Information about antecedents			
		6.2 Social comparison			
		6.3 Information about others’ approval			
		7.1 Prompts/cues			
		12.5 Adding objects			
Patel et al. (2017)	-Environmental context and resources	4.2 Information about antecedents	*Defaults and Salience*	Not possible	Yes
	-Memory attention and decision processes	7.1 Prompts/cues			
Presseau et al. (2018)	-Beliefs about capabilities	1.1 Goal setting behavior	*Messenger*	Not possible	No
	-Emotions	1.6 Discrepancy between behavior and goal			
	-Goals	1.2 Problem solving			
	-Memory attention and decision processes	4.1 Instruction on how to perform behavior			
		6.1 Demonstration of the behavior			
		8.7 Graded tasks			
		8.1 Behavioral practice/rehearsal			
		8.3 Habit formation			
		9.1 Credible source			
		10.8 Incentive outcome			
		12.5 Adding objects to the environment			
		15.1 Verbal persuasion about capability			
Sacarny et al. (2018)	-Beliefs about consequences	2.1 Monitoring of behavior without feedback	*Messenger, Incentives and Norms*	Partially	Yes
	-Social influences	2.2 Feedback on behavior			
		6.2 Social comparison			
		6.3 Information about others’ approval			
Shakespeare et al. (2019)	-Knowledge	5.1 Information about health consequences	*Messenger, Defaults and Salience*	Not possible	Yes
	-Memory attention and decision processes	6.3 Information about others’ approval			
		7.1 Prompts/cues			
		12.1 Restructuring the physical environment			
Yadav et al. (2019)	-Knowledge	Intervention 1:	*Messenger, Salience, Priming and Commitments*	Not possible	Yes
	-Memory attention and decision processes	1.9 Commitment			
	-Social influences	2.2 Feedback on behavior			
		3.1 Social Support (unspecified)			
		6.3 Information about others’ approval			
		7.1 Prompts/cues			
		12.5 Adding objects to the environment			
		Intervention 2:	*Messenger, Norms, Salience, Priming and Commitments*	Not possible	Yes
		1.9 Commitment			
		2.2 Feedback on behavior			
		3.1 Social Support (unspecified)			
		6.2 Social comparison			
		6.3 Information about others’ approval			
		7.1 Prompts/cues			
		12.5 Adding objects to the environment			

a Each number represents a behavior change techniques cluster, and the technique within that number (Michie et al., 2013). The clusters are as follows: 1 = Goals and Planning, 2 = Feedback and Monitoring, 3 = Social Support, 4 = Shaping Knowledge, 5 = Natural Consequences, 6 = Comparisons of Behavior, 7 = Associations, 8 = Repetition and Substitution, 9 = Comparisons of Outcomes, 10, Reward and Threat, 11 = Regulation, 12 = Antecedent, 13 = Identity, 14 = Scheduled Consequences, 15 = Self-Belief, 16 = Covert Learning.

b Completely = A sufficient array of techniques was applied to influence all identified determinants, Partially = Some identified determinates were not targeted by any recommended techniques, Not possible = No techniques are recommended to influence an identified determinant.

In the 15 included studies, 9 of the 14 TDF determinants were identified. The median number of determinants identified was 2 (range 1–4). As multiple determinants were identified in most studies, we could anticipate that multiple techniques would be recommended to influence behavior effectively. The ‘memory attention and decision processes’ was identified as a determinant in 12 studies, and this poses a potential problem for interventionists because there are no recommended techniques to address this determinant. No studies identified the “behavioral regulation,” “intentions,” “optimism,” “reinforcement,” and “skills” determinants.

### 3.5 Behavior Change Techniques

The behavior change techniques applied in each of the 20 interventions along with their clusters are provided in the third column in Table 3. Reviewers’ initial agreements were high (96%). Twenty-six of the 93 techniques were applied, which sit in 12 of the 16 clusters. The median number of techniques applied was 4 (range 1–12). Techniques from the Associations cluster (*n* = 13; e.g., “7.1 prompts/cues”) and the Antecedent cluster (*n* = 11; e.g., “12.1 restructuring the physical environment” beyond adding prompts/cues), were applied most, followed closely by techniques from the Shaping Knowledge cluster (*n* = 9; e.g., “4.1 instruction on how to perform behavior”). No interventions applied techniques from the Covert Learning, Regulation, Identity, and Scheduled Consequences clusters.

### 3.6 MINDSPACE Contextual Influencers Applied

The MINDSPACE contextual influencers applied in each intervention are provided in the fourth column of Table 3. Reviewers’ initial agreements were high (80%). The median number of contextual influencers applied was 2 (range 1–5). Of the nine contextual influencers, eight were applied across the interventions. The most often applied contextual influencer was *defaults* (*n* = 10). No interventions applied the *ego* influencer.

The *defaults* influencer was often applied by altering the organization’s prescribing software (*n* = 7). For example, Patel et al. (Patel et al., 2017) inserted default reminders to vaccinate all incoming patients against influenza, and Malhotra et al. (Malhotra et al., 2016) altered the search function to return generic medication options when brand name medications were entered.

The *commitments* influencer was often applied by asking prescribers to sign a document in studies to optimize antibiotic prescribing (*n* = 4). For example, in Meeker et al.’s (Meeker et al., 2014) intervention clinicians signed a personal poster-sized commitment letter featuring their photographs and signatures along with a stated commitment to avoiding inappropriate antibiotic prescribing. These personal commitments were displayed in the practitioners’ examination rooms for 12 weeks. In Mafi et al.’s (Mafi et al., 2018) and Yadav et al.’s (Yadav et al., 2019) interventions (both of which aimed to optimize antibiotic prescribing), the similar commitment documents were displayed in more public areas, such as waiting rooms. In Yadav’s intervention, some practitioners also wore badges signaling their commitment to better antibiotic stewardship.

The *norms* influencer was often applied by sending prescribers letters with peer-comparison information (*n* = 3). Patel et al.’s (Patel et al., 2018) and Yadav et al.’s (Yadav et al., 2019) intervention letters compared practitioners’ medication use to the 90th percentile performers for statins and antibiotics, respectively, and Sacarney et al.’s (Sacarny et al., 2018) intervention letters compared practitioner medication use to the 75th percentile performers for quetiapine.

Did the interventions apply a sufficient array of recommended techniques, and were they effective?

The fifth column of Table 3 describes whether the behavior change techniques applied in each intervention addressed all its study’s identified determinants. The last column describes whether each intervention was effective; 16 of the 20 interventions (80%) were effective.

Only two of the interventions applied a sufficient array of recommended techniques to address all identified determinants, and only one of these interventions was effective. Regarding the effective intervention, Isenberg et al. (Isenberg et al., 2018) noted that emergency department practitioners may not be aware of new guidelines published around pain management for mechanically ventilated patients and that workflow inadequacies may be preventing optimal analgesic and benzodiazepine use. To build practitioners’ knowledge, they provided feedback on their current prescribing and instructions about how to follow the new guidelines. To address workflow inadequacies, they started to stock analgesics in the emergency department and removed benzodiazepines from an existing order set.

Regarding the non-effective intervention, Lemiengre et al. (Lemiengre et al., 2018) identified “environmental contexts and resources,” “knowledge,” “emotions,” and “social influences” as determinants of pediatric practitioners’ inappropriate antibiotic use. They then used a factorial design to test the effectiveness of two intervention components, in isolation and combination, against a control condition where neither intervention component was applied. The first intervention simply provided practitioners with quick finger-prick diagnostic tests to mitigate uncertainty about the diagnosis. The second intervention provided practitioners with a supportive set of questions they could ask parents and a safety net brochure they could give parents to cope with parental pressure. The third intervention applied both intervention components to address all identified determinants but was not effective.

Three of the interventions applied techniques that partially addressed their identified determinants. Two were not effective and one was effective. The two that were not effective were Lemiengre et al.’s (Lemiengre et al., 2018) single component interventions described above. The other intervention was Sacarny et al. (Sacarny et al., 2018), for which they identified “social influences” and “beliefs about consequences” as behavioral determinants of practitioners’ suboptimal quetiapine fumarate use. Their intervention involved sending practitioners letters that compared their prescribing to their peers’ prescribing (a social influence), but they did not include additional information to emphasize the natural consequences of poor prescribing practices.

The remaining 15 interventions could not address all their identified determinants, because there are no recommended techniques to address the “memory, attention, and decision processes” or “social/professional role and identity” determinants. Yet, these interventions were still effective. In these interventions, the contextual influencers may have bolstered the effectiveness of the intervention. Of these 15 interventions, the most frequently applied contextual influencers were the *defaults* influencer (*n* = 9), followed by the *salience* influencer (*n* = 7), *norms* and *messenger* (*n* = 5), *commitments* (both *ns* = 4), and *priming* (*n* = 3). Note that as each intervention could apply more than one contextual influencer, these numbers do not sum to 15. While space does not permit an extensive review of all the 15 interventions here (see Supplementary Appendix 3 for additional information), two of the effective interventions that creatively altered the existing choice environment are described below.

Harewood et al.’s (Harewood et al., 2011) study identified “environmental resources and contexts” and “memory, attention, and decision processes” as behavioral determinants of suboptimal midazolam use for endoscopic sedation. Their intervention involved pre-filling syringes with different amounts of midazolam. This intervention could be described as simply applying the “restructuring the physical environment” technique that is well suited to overcome the “environmental resources and context” determinant. However, we posit that the *defaults* and *priming* influencers may have bolstered the effectiveness of this intervention, as prescribers’ medication use trended towards the pre-filled amounts labeled on the syringe in a manner anticipated by the literature on default and anchoring effects (Tversky and Kahneman, 1974; Johnson and Goldstein, 2003).

As another example (Musgrove et al., 2018), identified “knowledge” and “memory attention and decision processes” as behavioral determinants of suboptimal antibiotic use for hospital in-patients. Before their intervention when there was no indication of a bacterial infection, the results of the diagnostic tests given to clinicians stated that the sample contained “commensal respiratory flora.” Their intervention modified the language in this report to emphasize that there was “commensal respiratory flora only: No *S. aureus*/MRSA or *P. aeruginosa*.” This intervention could be described as using a technique to address only the “knowledge” determinant. However, as clinicians likely already had sufficient knowledge to understand the initial reporting language, enhancing the *salience* of the “no” bacteria information more likely contributed to this intervention’s beneficial effect.

## 4 Discussion

The current systematic review included 15 studies describing interventions that aim to optimize prescribing decisions without forbidding options or changing economic incentives. Of the 20 interventions described in these 15 studies, 16 (80%) were effective. Regarding the review’s first objective, to describe the behavior change techniques and contextual influencers applied, 26 behavior change techniques and 8 contextual influencers were applied. Regarding the second objective, to describe whether the previous interventions applied a sufficient array of recommended techniques, only two did. As so few interventions applied recommended techniques, there is insufficient information to conclude whether interventions that apply recommended techniques are more effective than those that do not.

The current findings echo other reviews’ findings that behavioral interventions can optimize prescribing. For example, a systematic review of 31 randomized controlled trials finds that medication reviews can reduce the number of medication-related problems patients experience, and the number of medications ultimately prescribed (Huiskes et al., 2017). Beyond improving patients’ wellbeing, Hasan et al.’s economic analysis notes that medication reviews can also result in significant cost savings to support the financial stability of healthcare organizations (Hasan et al., 2017). Another systematic review finds that computerized clinical decision support systems improved care performance in 37 of the 59 studies examined (Hemens et al., 2011). However, the positive effects of computerized clinical decision support systems interventions on patient outcomes are less reliable, as only 6 of the 29 trials that assessed patient outcomes in their review found improvements.

To the authors’ knowledge, this is the first study to use TDF, BCTs Taxonomy version 1, and MINDSPACE framework at once to characterize behavioral change interventions. Coding the interventions according to the TDF, BCTs Taxonomy, and MINDSPACE framework allows the current review to comment not only on those intervention techniques attempted, but to highlight some techniques that have not yet been evaluated. This is of course not to say that unevaluated options will work but to emphasize that absence of evidence does not entail it is not effective (Altman and Bland, 1995). For example, several studies identified “social influences” as a behavioral determinant of suboptimal prescribing. The “social influences” determinant can be influenced by techniques in the Identity cluster, but none of these studies applied techniques from the Identity cluster (e.g., “13.1 identification of self as a role model”). In addition, several studies identified “emotions” as a behavioral determinant. The “emotions” determinant can be influenced by techniques in the Regulation cluster, but none of these studies applied techniques from the Regulation cluster (e.g., “11.2 reduce negative emotions”). Regarding the MINDSPACE contextual influencers, the *ego* influencer was never applied. For *ego*, interventions that emphasize the reputational benefits favoring certain prescribing decisions might prove effective where “social/professional role and identity” is identified as a determinant of suboptimal prescribing, e.g., around publicized low-value services (Bishop et al., 2017).

The recommended linkages provided by Cane et al. (Cane et al., 2015) were used in the current review because these linkages ensure that all 93 behavior change techniques are linked to at least one TDF domain. Without using the clusters, some behavior change techniques are missing links to the TDF domains. The Behavior Change Techniques Taxonomy version 1 is the most exhaustive list of behavioral change techniques, but the Theoretical Domains Framework is not the most exhaustive list of potential behavioral determinants. Recently the 14 domains have been joined up with 12 of the most frequently cited mechanisms of action, e.g., “self-image” and “values” to yield a more extensive list of 26 determinants. These 26 determinants have been linked to only 70 behavior change techniques *via* a literature review (Carey et al., 2019) and 61 techniques have been linked *via* an expert consensus study (Connell et al., 2019). An interactive tool, called the Theory and Techniques Tool, has been created to bring these more recent findings together. As of May 2021, this tool provides researchers with links between 74 behavior change techniques and the 26 determinants (https://theoryandtechniquetool.humanbehaviorchange.org/).

Within the Theory and Techniques Tool, the “memory attention and decisions processes” is linked with high certainty to the “7.1 prompts/cues” technique. In the current review, 12 studies identified ‘memory attention and decision processes’ as a determinant, and 9 applied the “7.1 prompts/cues technique.” Therefore, applying these new connections reveals a great deal more studies where the techniques aligned with the determinants identified. However, the “social/professional role and identity” determinant is still not conclusively linked to any of these 74 behavior change techniques. Additionally, those 19 techniques missed off the original lists (93 − 74 = 19) will not be linked up to any of the determinants in the expanded list.

Some limitations of the current review are now noted. First, the current review includes articles published before July 2019, which will be beyond the recommended 2 years when this article is published. As research around nudge interventions has likely expanded since Thaler and Sunstein published their book “Nudge,” and since Thaler’s won the Nobel prize in economics in 2017 (Earl, 2018), new articles may have been missed from this review. While the present synthesis of previous studies can inform future research and enhance methods of future literature reviews, researchers and policymakers could seek more recent evidence to inform their studies and changes in practice. Second, the search only includes articles that have been published in peer-reviewed journals in the English language, and this may exacerbate the negative effects of publication biases. A future review may increase the scope of the current findings by including articles published in non-English languages, and in the grey literature (e.g., policy documents or internal organizational reports). Third, nearly half of the studies in our review made use of non-randomized methods, which have a greater likelihood for bias than randomized methods.

A fourth limitation is that behavioral determinants are often vaguely reported in published intervention studies and the behavior change techniques are not easy to code some may have been excluded (Abraham et al., 2015). The high percentage agreements in the intervention coding are in part a function of the large number of determinants, techniques, and contextual influencers that the reviewers could easily say were not being used. To improve the reporting of interventions, we urge researchers to describe their interventions using the TIDieR checklist (Hoffmann et al., 2014). We also urge researchers to identify the techniques they apply using the BCT Taxonomy (Michie et al., 2013) and to consider what other influences are at play, particularly those developed in behavioral economics.

In conclusion, the current review included 15 studies with 20 interventions of which 16 were effective. This encouraging finding supports the development of future behavior change interventions to optimize prescribing, which do not include mandates or significantly alter financial incentives. The present review encourages interventionists to understand the behavioral determinants they are trying to affect, before selecting techniques/contextual influencers to apply. The TDF, BCTs Taxonomy version 1, and MINDSPACE framework are three theoretically informed tools that interventionists can consider when developing complex interventions to optimize prescribing and ultimately improve patient wellbeing.

## Data Availability

The raw data supporting the conclusions of this article will be made available by the authors, without undue reservation.
