# Supplementary material for: A Systematic Review of Nudge Interventions to Optimize Medication Prescribing
Source: Front Pharmacol. 2022 Jan 25;13:798916. doi: 10.3389/fphar.2022.798916 (PMC8822212; doi:10.3389/fphar.2022.798916)
Supplement: Supplementary file 2 [file DataSheet1.docx]

Appendix 1*.* Information about the eligibility criteria and search strategy.

Eligibility Criteria: PICOS criteria for inclusion and exclusion

|  | Inclusion criteria | Exclusion criteria |
| --- | --- | --- |
| Participants | Medical and non-medical staff in healthcare settings, including but not limited to consultants, general practitioners, junior doctors, pharmacist prescribers, nurse prescribers, dentists, etc. | Assistant health workers, community medical assistants and practitioners without legal rights to prescribe as reported in some countries. |
| Interventions | All nudge and low-cost, light-touch behavioral intervention that aimed to optimize medication prescribing, or to improve cost effectiveness of prescribing. Such intervention will consider context variation with behavioral determinants, wherein nudged prescriptions are included. This also adds studies at the behavioral and cognitive level, where interventions may focus on addressing heuristics, habits and impulses – that deviate from optimal prescription responses. For example, a doctor may prescribe medication, because of the effects of Priming or Messenger.  Our review of interventions targeting optimal prescribing will draw on studies of interventions that utilize behavioral change techniques from behavioral economics found in frameworks such as MINDSPACE and systematic reviews of cognitive biases and heuristics. | The intervention that are proposed through theory but not implemented and evaluated. The included studies needed to discuss both behavioral determinants and behavior change techniques: studies that only addressed one of these components were excluded. |
| Comparisons | Prescribers receiving no intervention, prescribers receiving intervention, other than ‘nudges’ and ‘low-cost light-touch behavioral intervention’ | Not applicable |
| Outcomes | Primary outcomes: prescribing behaviors indicators (e.g. adherence to treatment guidelines, number of medicines, medicine switches, patient health, costs, and material waste (i.e., dispensed but unused medications).  Secondary: none | Outcomes other than medicines (e.g. improvement in utilisation of surgical items, change in number of diagnostic tests referred, etc.) |
| Study designs | Randomized controlled trials, quasi-experimental studies, cohort studies, case control studies, controlled before-after studies. For controlled before-after studies, we look for multiple sites or units to accommodate intervention groups and control groups. | Case studies, reviews, editorials, letters or conference abstracts (unless providing sufficient data to be included in the review) |

Information sources and Search Strategy: Search strategy in Medline (Ovid)

|  | Search terms |
| --- | --- |
| 1 | Nudg*.mp. [mp=title, abstract, original title, name of substance word, subject heading word, floating sub-heading word, keyword heading word, organism supplementary concept word, protocol supplementary concept word, rare disease supplementary concept word, unique identifier, synonyms] |
| 2 | (choice adj1 architect*).mp. [mp=title, abstract, original title, name of substance word, subject heading word, floating sub-heading word, keyword heading word, organism supplementary concept word, protocol supplementary concept word, rare disease supplementary concept word, unique identifier, synonyms] |
| 3 | (theoretical adj1 domain adj1 framework*).mp. [mp=title, abstract, original title, name of substance word, subject heading word, floating sub-heading word, keyword heading word, organism supplementary concept word, protocol supplementary concept word, rare disease supplementary concept word, unique identifier, synonyms] |
| 4 | (behavio?r adj1 change adj1 (wheel* or technique* or intervention* or process* or method* or strateg*)).mp. [mp=title, abstract, original title, name of substance word, subject heading word, floating sub-heading word, keyword heading word, organism supplementary concept word, protocol supplementary concept word, rare disease supplementary concept word, unique identifier, synonyms] |
| 5 | 1 or 2 or 3 or 4 |
| 6 | prescrib*.mp. [mp=title, abstract, original title, name of substance word, subject heading word, floating sub-heading word, keyword heading word, organism supplementary concept word, protocol supplementary concept word, rare disease supplementary concept word, unique identifier, synonyms] |
| 7 | prescript*.mp. [mp=title, abstract, original title, name of substance word, subject heading word, floating sub-heading word, keyword heading word, organism supplementary concept word, protocol supplementary concept word, rare disease supplementary concept word, unique identifier, synonyms] |
| 8 | medic*.mp. [mp=title, abstract, original title, name of substance word, subject heading word, floating sub-heading word, keyword heading word, organism supplementary concept word, protocol supplementary concept word, rare disease supplementary concept word, unique identifier, synonyms] |
| 9 | 6 or 7 or 8 |
| 10 | 5 and 9 |

Appendix 2. List of excluded articles (after full text review) with reasons for exclusions.

| Reference | Reason for exclusion |
| --- | --- |
| Arnold et al. 2011 (1) | The outcome measured was not medicine prescription, the study measured the use of frozen plasma |
| Bourdeaux et al. 2016 (2) | The outcome measure was not medicine prescription, the study measured the tidal volume of ventilation |
| Brodrick 2015 (3) | The outcome measure was not medicine prescription, the study measured the rates of CPR and escalation decision-making for oncology inpatients |
| Cadogan et al. 2015 (4) | The study did not report about the evaluation of the proposed intervention. |
| Creupelandt et al 2017 (5) | The study did not report about the evaluation of the proposed intervention in an objective way. |
| Finkelstein et al. 2008 (6) | The intervention was not exclusively targeted to prescribers, patients were also targeted |
| Francis et al. 2009 (7) | The study did not report about the evaluation of the proposed intervention. |
| Goulding et al. 2015 (8) | The study did not report about the evaluation of the proposed intervention. |
| Hrisos et al. 2008 (9) | This study was a simulated, the planned intervention did not measure medicine prescription in real world scenario. |
| Kavookjian and Mamidi 2008 (10) | The study identified prescribers’ motivations and barriers for prescribing medicine, the researchers did not developed or evaluated any intervention. |
| Kim et al. 2018 (11) | Insufficient information was provided to describe the active choice intervention. |
| King et al. 2014 (12) | This study was a simulated, the planned intervention did not measure medicine prescription in real world scenario. |
| Koh et al. 2017 (13) | The intervention was targeted to patients rather than prescribers; and the study also did not involve the measurement of medicine use, instead measure the number of completed physical exercises |
| Liao and Navathe 2018 (14) | The study did not report the evaluation of the intervention, rather it was editorial description of medication use problem and the potential solution. |
| Mafi et al. 2016 (15) | The outcome measured was not medicine prescription, the study measured use of diagnostic imaging services. |
| Murphy et al. 2017 (16) | The study did not report about the evaluation of the proposed intervention. |
| Ng, Le Couteur, and Hilmer 2018 (17) | The intervention was not exclusively targeted to prescribers, patients and pharmacists were also targeted. The study did not report about the evaluation of the proposed intervention. |
| Patel et al. 2018 (18) | Conference abstract of the article Patel et. al 2018, one of the included studies in this review. |
| Patel et al. 2016 (19) | Conference abstract of the article Patel et. al 2016, one of the included studies in this review. |
| Pollmann et al. 2015 (20) | The study did not propose any intervention, this was scoping review of potential methods to de-prescribe Benzodiazepines and other Z medicines |
| Probst, Shaffer, and Chan 2013 (21) | The outcome measured was not medicine prescription, the study measured the laboratory tests |
| Tannenbaum et al. 2015 (22) | This study was a simulated, the planned intervention did not measure medicine prescription in real world scenario. |
| Treweek et al. 2013 (23) | This study was a simulated, the planned intervention did not measure medicine prescription in real world scenario. |
| Treweek et al. 2016 (24) | The study did not report about the evaluation of the proposed intervention. |
| Treweek et al. 2011 (25) | The study did not report about the evaluation of the proposed intervention. |
| Warburton et al. 2019 (26) | Conference abstract, did not report about the evaluation of the proposed intervention. |

References for excluded studies

1. Arnold DM, Lauzier F, Whittingham H, Zhou Q, Crowther MA, McDonald E, Cook DJ. A multifaceted strategy to reduce inappropriate use of frozen plasma transfusions in the intensive care unit. J Crit Care. 2011;26(6):636.e7-636.e13. https://doi.org/10.1016/j.jcrc.2011.02.005

2. Bourdeaux CP, Thomas MJ, Gould TH, Malhotra G, Jarvstad A, Jones T, Gilchrist ID. Increasing compliance with low tidal volume ventilation in the ICU with two nudge-based interventions: evaluation through intervention time-series analyses. BMJ Open. 2016;6(5):e010129. https://doi.org/10.1136/bmjopen-2015-010129

3. Brodrick R. EP-1284: Nudge theory: a cost-effective method for increasing resuscitation decision-making in oncology inpatients. Radiat. Oncol. 2015;115(1):S693 https://doi.org/ 10.1016/S0167-8140(15)41276-9

4. Cadogan, C.A., Ryan, C., Francis, J.J. Gormley GJ, Passmore P, Kerse N, Hughes CM. Improving appropriate polypharmacy for older people in primary care: selecting components of an evidence-based intervention to target prescribing and dispensing. Implement Sci. 2015;10(1):161. https://doi.org/10.1186/s13012-015-0349-3

5. Creupelandt H, Anthierens S, Habraken H, Declercq T, Sirdifield C, Siriwardena AN, et al. Teaching young GPs to cope with psychosocial consultations without prescribing: a durable impact of an e-module on determinants of benzodiazepines prescribing. BMC Med Educ. 2017;17(1):259. https://doi.org/10.1186/s12909-017-1100-3

6. Finkelstein JA, Huang SS, Kleinman K, Rifas-Shiman SL, Stille CJ, Daniel J, Schiff N, Steingard R, Soumerai SB, Ross-Degnan D, Goldmann D, Platt R. Impact of a 16-community trial to promote judicious antibiotic use in Massachusetts. Pediatrics. 2008;121(1):e15‐e23. https://doi.org/doi:10.1542/peds.2007-0819

7. Francis JJ, Stockton C, Eccles MP, Johnston M, Cuthbertson BH, Grimshaw JM, Hyde C, Tinmouth A, Stanworth SJ. Evidence-based selection of theories for designing behavior change interventions: using methods based on theoretical construct domains to understand clinicians' blood transfusion behavior. Br J Health Psychol. 2009;14:625-46. https://doi.org/10.1348/135910708x397025

8. Goulding L, Parke H, Maharaj R, Loveridge R, McLoone A, Hadfield S, Helme E, Hopkins P, Sandall J. Improving critical care discharge summaries: a collaborative quality improvement project using PDSA. BMJ Qual Improv Rep. 2015;4(1):u203938.w3268. https://doi.org/10.1136/bmjquality.u203938.w3268

9. Hrisos S, Eccles M, Johnston M, Francis J, Kaner, EFS, Steen N, Grimshaw J. An intervention modelling experiment to change GPs' intentions to implement evidence-based practice: using theory-based interventions to promote GP management of upper respiratory tract infection without prescribing antibiotics #2. BMC Health Serv Res. 2008;8(1):10. https://doi.org/10.1186/1472-6963-8-10

10. Kavookjian J, Mamidi S. Prescribing of beta-blockers after myocardial infarction: a preliminary study of physician motivations and barriers. Clin Ther. 2008;30(pt2):2241‐9. https://doi.org/10.1016/j.clinthera.2008.12.006

11. Kim RH, Day SC, Small D, Rareshide C, Patel M. Change in influenza vaccination rates by time of day and after an active choice intervention in the electronic health record. J Gen Intern Med. 2018;33(Supplement 2):S136-7.

12. King D. Jabbar A. Charani E, Bicknell C, Wu Z, Miller G, Gilchrist M, Vlaev I, Franklin BD, Darzi A. Redesigning the ‘choice architecture’ of hospital prescription charts: a mixed methods study incorporating in situ simulation testing. BMJ Open 2014;4(12):e005473. http://dx.doi.org/10.1136/bmjopen-2014-005473

13. Koh LH, Hagger MS, Goh VHH, Hart WG, Gucciardi DF. Effects of a brief action and coping planning intervention on completion of preventive exercises prescribed by a physiotherapist among people with knee pain. J Sci Med Sport. 2017;20(8):723‐8. https://doi.org/10.1016/j.jsams.2017.02.008

14. Liao, J. M., and A. S. Navathe. Nudging physicians to reduce quetiapine prescribing using Medicare letters: following the letters of the law? JAMA Psychiatry. 2018;75(10):989-90. https://doi.org/10.1001/jamapsychiatry.2018.1843

15. Mafi, J. N., J. N. Sayles, M. K. Patel, D. Kahaku, C. A. Carillo, J. F. Brunner, A. Mahajan, L. Sarff, S. Ettner, K. L. Kahn, and C. Sarkisian. 2016. Implementing an electronic clinical decision support tool to reduce low value imaging for back pain INA large safety net health system. J Gen Intern Med. 2016;31(Supplement 2): S268.

16. Murphy ME, Byrne M, Zarabzadeh A, Corrigan D, Fahey T, Smith SM. Development of a complex intervention to promote appropriate prescribing and medication intensification in poorly controlled type 2 diabetes mellitus in Irish general practice. Implement Sci. 2017;12(1):115. https://doi.org/10.1186/s13012-017-0647-z

17. Ng BJ, Le Couteur DG, Hilmer SN. Deprescribing benzodiazepines in older patients: impact of interventions targeting physicians, pharmacists, and patients. Drugs Aging. 2018;35(6):493‐521. https://doi.org.10.1007/s40266-018-0544-4.

18. Patel M, Kurtzman GW, Kannan S, Small D, Morris A, Honeywell S, Leri D, Rareshide C, Day SC, Mahoney K, Volpp KG, Asch DA. Effect of an automated patient dashboard using active choice and peer comparison performance feedback to physicians on statin prescribing: The pre-scribe randomized clinical trial. J Gen Intern Med. 2018;33(Supplement 2): S174-5.

19. Patel M, Volpp KG, Small D, Zhu J, Yang L, Honeywell S, Day SC. Using active choice within the electronic health record to increase influenza vaccination rates. J Gen Intern Med. 2016;31(Supplement 2):S456.

20. Pollmann AS, Murphy AL, Bergman JC, Gardner DM. Deprescribing benzodiazepines and Z-drugs in community-dwelling adults: a scoping review. BMC Pharmacol Toxicol. 2015;16(1):19. https://doi.org/10.1186/s40360-015-0019-8

21. Probst CA, Shaffer VA, Chan Y. The effect of defaults in an electronic health record on laboratory test ordering practices for pediatric patients. Health Psychol. 2013;32(9):995-1002. https://doi.org/10.1037/a0032925

22. Tannenbaum D, Doctor JN, Persell SD, Friedberg MW, Meeker D, Friesema EM, et al. Nudging physician prescription decisions by partitioning the order set: results of a vignette-based study. J Gen Intern Med. 2015;30(3):298-304. https://doi.org/10.1007/s11606-014-3051-2

23. Treweek S, Bonetti D, Barnett K, Eccles M, Francis J, Jones C, MacLennan G, Pitts N, Ricketts I, Sullivan F, Weal M. A92: Web-based intervention modelling experiments: A way of exploring professional behavior change interventions before a full-scale trial. Clinical Trials. 2013;10(Supplement 2):S42-3.

24. Treweek S, Francis JJ, Bonetti D, Barnett K, Eccles MP, Hudson J, Jones C, Pitts NB, Ricketts IW, Sullivan F, Weal M, MacLennan G. A primary care Web-based Intervention Modeling Experiment replicated behavior changes seen in earlier paper-based experiment. J Clin Epidemiol. 2016;80:116‐122. doi:10.1016/j.jclinepi.2016.07.008

25. Treweek S, Ricketts IW, Francis J, Eccles M, Bonetti D, Pitts NB, Maclennan G, Sullivan F, Jones C, Weal M, Barnett K. Developing and evaluating interventions to reduce inappropriate prescribing by general practitioners of antibiotics for upper respiratory tract infections: a randomized controlled trial to compare paper-based and web-based modelling experiments. Implement Sci. 2011;6(1):16. https://doi.org/10.1186/1748-5908-6-16

26. Warburton J, Jones N, Cooper R, Turner P, Bourdeaux C. Validating the hierarchy of intervention effectiveness for prescribing nudges in critical care. Critical Care. 2019. Conference: 39th International Symposium on Intensive Care and Emergency Medicine. 19-22 March 2019, Brussels, Belgium.

Appendix 3. A description of each evaluated intervention following the TIDieR checklist

| **Study** | **Name**  Name that describes intervention | **Why**? Rationale, goal of intervention, and theory. | **What**?  Materials used | **What**?  Procedures used | **Who**?  Person who delivered the intervention | **How**?  How the intervention was delivery (e.g. face-to-face) | **Where**?  The immediate setting of the intervention (e.g. in office) | **How much?**  The number of times and/or duration the intervention was delivered | **Tailoring**?  Describe any personally tailored features of the intervention | **Modifications**?  Describe any change to the intervention during the trial. | **Planned implementation fidelity**?  Who assessed the interventions implementation fidelity and how | **Actual implementation fidelity**?  Was the intervention implemented as planned? |
| --- | --- | --- | --- | --- | --- | --- | --- | --- | --- | --- | --- | --- |
| Bourdeaux et al (2014) – Intervention 1 | Adding defaults to a computerized prescribing template: Chlorhexidine | Past research suggests that defaults can influence choice-behavior.  This intervention arm of the study assesses the effectiveness of adding a medication to a computerized template to increase use of chlorhexidine for patient ventilated with pneumonia.  Based on theories from of Behavioral economics and Nudge. | An electronic-prescribing system. | Intervention 1 – Added chlorhexidine as a default in the prescribing template. | Not clear who initiated the change to the electronic-prescribing system. | A computer interface. | Intensive Care Unit | The intervention was active from January 2010 to November 2012 | Not applicable | No modifications reported | Information not provided | Information not provided |
| – Intervention 2 | Removing defaults from a computerized prescribing template: Hydroxyethyl starch (HES) | See ‘rationale’ and ‘theory’ above.  This intervention arm of the study assesses the effectiveness of removing a medication from a computerized template to decrease use of HES for patient ventilated with pneumonia. | See above column | Intervention 2 – Removed HES as a default in the prescribing template. | See above column | See above column | Intensive Care Unit | The intervention was active from April 2009 to September 2012 | See above column | See above column | See above column | See above column |
| Harewood et al (2011) | Size matters: changing the Midazolam dispensing unit | The minimum amount of Midazolam needed for endoscopic sedation is not always used.  This study assesses the effectiveness of decreasing how much Midazolam is preloaded into the dispensing unit (3ml or 5ml) to decrease Midazolam use.  The principles of behavioral economics were adopted. | Different syringes sizes (5ml and 3ml) prefilled with Midazolam. | Intervention – Midazolam was prefilled in either 3ml or 5ml syringes. Endoscopists could use as many prefilled syringes as they felt necessary, and did not have to empty syringes. | It is unclear who made the syringes available.  The endoscopists prefilled the syringes. | Different syringes were made available. | Hospital | The intervention was active form June 2010 to July 2010 (8 weeks), with the size of the syringe altered each week between the 3ml syringe and the 5ml syringe size. | Not applicable | No modifications reported | Information not provided | Information not provided |
| Isenberg et al (2018) | Education + physical and virtual environmental adjustments: Analgesics and benzodiazepines | The Society for Critical Care Medicine’s (SCCM) guidelines to support mechanically ventilated patients are not followed sufficiently.  This study assesses effectiveness of an educational intervention, complimented by two simple changes in workflow, to increase guideline compliance: increase analgesic use and decrease benzodiazepine use.  The principles of behavioral economics underpin the intervention – designed to nudge behavior. | Information about SCCM guidelines and current practice.  Medication dispensing units in emergency department.  An electronic-prescribing system. | Intervention – A large educational campaign took place targeting emergency physicians and nurses. Emergency physicians received emails and attended faculty meetings about SCCM guidelines that included information about current practice and recommendations for future practice. Nurses saw presentations about SCCM guidelines online, and the intervention team met them to discuss the initiative.  In addition to education, two workflow changes took place. First, fentanyl infusions were stocked in the emergency department (rather than in central pharmacy) to reduced waiting times. Second, the computerized prescribing template was adjusted such that physicians could easily order fentanyl and propanol together, but to order benzodiazepines they had to use a search function. | The authors who are also emergency physicians. | Education intervention component: Face-to-Face meetings, emails, and online presentations.  Environmental intervention component: Adding fentanyl infusions to medication dispensing units in the department, and the computerized prescribing template. | Emergency Department | The intervention was active from 2015 to 2016. | Not applicable | No modifications reported | Information not provided | Information not provided |
| Lemiengre et al (2018) – Intervention 1 | Finger-prick test for antimicrobial infections | Antibiotics are overprescribed in primary care at least in part because physicians are uncertain about why children are ill (e.g. bacterial or viral).  This intervention arm of the study assesses effectiveness of making a diagnostic test available to decrease uncertainty and so decrease antibiotic prescribing.  No information about theory. | Point-of-care C-reactive protein test. | Intervention 1 – A point-of-care reactive protein test was made available for physicians to use that provided diagnostic information with within minutes. | It is unclear who made the tests available. | It is unclear how the tests were made available. | Primary Care | The intervention was active from February 2013 to February 2014. | Not applicable | No modifications reported | Information not provided | Information not provided |
| – Intervention 2 | Elicit parental concern combined with safety net advice around antibiotic use | Antibiotics are overprescribed in primary care, at least in part because of physicians’ failure to cope with parental concern (e.g. wanting to reassure parents when their child is ill).  This intervention arm of the study assesses effectiveness of a brief intervention that acknowledges and helps physicians cope with parental concerns to decrease antibiotic prescribing.  No information about theory. | Three questions.  Information leaflet. | Intervention 2 – Physicians asked parents the following three questions: Q1: ‘Are you concerned [about the illness of your child]?’ Q2: ‘What exactly concerns you?’, and Q3: ‘Why does this concern you?’ After hearing and responding to parents, physicians gave parents a safety net leaflet containing information about when to seek further help as safety advice. | It is unclear who directed the physicians to ask questions or to hand out the leaflets. | It is unclear how the questions and leaflets were made available. | See above column | See above column | See above column | See above column | See above column | See above column |
| - Intervention 3 | Finger-prick test + Elicit parental concern combined with safety net advice around antibiotic use | See ‘rationale’, ‘theory’ and ‘goal’ in above two columns. | Point-of-care C-reactive protein test.  Three questions.  Information leaflet. | See above two columns | See above two columns | See above two columns | See above column | See above column | See above column | See above column | See above column | See above column |
| Mafi et al (2018) | Education + Commitments to change: Antibiotics | Prescription rates of low-value antibiotics for uncomplicated bronchitis remain too high.  This study assesses the effectiveness of a multi-part intervention aimed to decrease such antibiotic use.  Based on the principles of behavioral economics, to nudge behavior. | Emails.  Past prescription records.  Posters.  Prescription pads. | Intervention – This intervention has four parts. The medical director emailed clinicians (1) the antibiotic guidelines, and (2) feedback about their previous antibiotic prescribing. Clinicians were also asked to (3) sign a large poster to display in the clinic stating their commitment to avoid prescribing low value antibiotics for bronchitis and (4) were provided with prescription pads reminding them to offer non-antibiotic treatment to patients. | The authors who are also clinicians. | Email, face-to-face interactions and the post | Emergency Department and Primary Care sites | The intervention was active from October 2016 to February 2017. | Not applicable | No modifications reported | Information not provided | Information not provided |
| Malhotra et al (2016) | Changing search function defaults on a computerized prescribing system: Generics medications | Branded medications tend to cost more than generic medications, and hospitals should reduce unnecessary costs where possible.  This study assesses the effectiveness of changing default medications on a computerized template to increase use of generic medications.  Adopted ideas from behavioral economics; specifically, default option setting (i.e. nudging). | An electronic-prescribing system. | Intervention – The e-prescribing system’s search function was redesigned such that when physicians searched for a brand-name medication, they were presented with options to prescribe that brand name medication or its generic equivalents. If the physicians selected a generic option, they only needed to click that option. If the physicians selected a brand option, they needed to click that option and then click to confirm that they intended to prescribe that branded option as opposed to its generic. | The authors who are also doctors and e-prescribing implementation staff. | Computer interface | Ambulatory Multispecialty Practice | The intervention was active from November 2019 to January 2020. | Not applicable | Not applicable | Information not provided | Information not provided. |
| Meeker et al (2014) | Commitments to change: Antibiotics | Poor antibiotic prescribing for acute respiratory infections persists in part due to defensive prescribing, poor awareness of guidelines, patient demand and psychosocial factors like cultural tendencies.  This study assesses the effectiveness of clinicians signing a public commitment to improve antibiotic prescribing.  This intervention was based on the principles public commitment. | Posters. | Intervention – Clinicians were asked to sign a commitment poster with a photograph of themselves and a statement saying that they would avoid inappropriate antibiotic prescribing. Clinicians were asked to display their commitment poster in their examination rooms. | The authors, which include the Clinical Research Informatics, program within the Southern California Clinical and Translational Science Institute. | Displayed posters in examination rooms. The content of the posters was written in the English and Spanish language. | Primary Care | The intervention was active for one year.  Clinician were asked to display their commitment poster for 12-weeks. | Not applicable | No modifications reported | Information not provided | Information not provided |
| Musgrove et al (2018) | Clearer language in diagnostic test results for antimicrobial agents | Part of the reason for the overuse of antibiotics in hospital may be the language microbiologists use to report the results of a test back to physicians.  This study assesses the effectiveness of changing the clarity of the language microbiologists use to decrease antibiotic medication use.  The intervention was based on the behavioral strategies to improve clarity of reporting, rooted in ideas from behavioral economics. | Microbiology reports. | Intervention – Before the intervention, microbiologists reports would tell physicians if a sample contained “commensal respiratory flora”, and possibly it was not clear that this meant there was no *S. aureus*/MRSA or *P. aeruginosa*. This reporting language was modified to clarify that no diagnostically relevant antimicrobial agents were present, with the phrase: “commensal respiratory flora only: No *S. aureus*/MRSA or *P. aeruginosa.”* | Microbiologists writing diagnostic test reports. | Linguistic modification made to statements in microbiology reports. | Hospital | The intervention was active from August 2016 to January 2017. | Not Applicable | No modifications reported | Information not provided | Information not provided |
| O’Connor (2009) | Using order sets to improve deep vein thrombosis (DVT) prophylaxis rates | DVT prophylaxis continues to be significantly underused in hospitalized patients.  This study assesses the effectiveness of providing pre-filled order sets to increase DVT prophylaxis use.  No theory is used. | Paper-based order sets. | Intervention – Before the intervention, physicians wrote all admission orders by hand. The intervention made pre-filled order sets available in the emergency department. In the DVT prophylaxis section of the order set, physicians could choose to order either 5,000 units of twice daily or compression stockings, or both, or request another form of DVT prophylaxis by hand in a free-text space. | The study authors. | Printed order sets were placed in the emergency department that could be re-ordered from the print shop. | Hospital | The intervention was active from December 2003 to March 2005. | Not applicable | No modifications reported | Information not provided | The availability of the order sets  was not formally assessed. |
| Patel et al (2018) – Intervention 1 | Soliciting active prescribing choices given an easy-to-use online dashboard: Statins | Half of patients that could benefit from statins are not prescribed statins.  This intervention arm in the study assesses the effectiveness of making information about patients eligible for statins more available to increase statins use.  The intervention was designed based on the principles of behavioral economics (i.e. nudge and active choice framing theory). | Emails.  Past prescription records.  Automated online dashboard. | Intervention 1 – Physicians were emailed a link to an automated online dashboard listing their patients who met national guidelines for statin therapy, but who had not been prescribed statins. Then they were asked to make an active choice: (a) to prescribe atorvastatin, (b) to prescribe another statin or (c) not to prescribe a statin and select a reason why not. | The study authors, including the Director of the Penn Medicine Nudge Unit. | Emailed link to automated online dashboard. | Primary Care | The dashboard was available from February 2017 to April 2017. | Not applicable | No modifications reported | Information not provided | Information not provided |
| Intervention 2 | Soliciting active prescribing choices given an easy-to-use online dashboard and Peer comparisons: Statins | This intervention arm in the study assesses the effectiveness of making information about patients eligible for statins easier to access AND peer comparisons about what other physicians are doing to increase statins use.  See ‘rationale’ and ‘theory’ in the above column. | See above column | Intervention 2 – In addition to the information provided in the above column, the email sent to physicians in this arm also included feedback on their previous statin prescribing. Prescribers below the median prescribing rate were informed of how they compared with the median. Prescribers above the median but below the 90^th^ percentile were informed how they compared with the 90^th^ percentile. Prescribers at or above the 90^th^ percentile were told that they were a top performer. | See above column | See above column | See above column | See above column | Information in letter tailored such that prescribers would learn about their own prescribing practices relative to peers. | See above column | See above column | See above column |
| Patel et al (2017) | Soliciting active vaccination choices given electronic health care: Influenza vaccinations | More than half of adults in the United States are not vaccinated against influenza.  This study assesses the effectiveness of automatically alerting clinicians to arriving patients who have not been vaccinated and prompting those clinicians to make an activity choice to do so.  Ideas about active choice intervention from behavioral economics were adopted. | Electronic Health records.  Computerized prompts. | Intervention – The electronic health record was used to alert clinics that a patient had not been vaccinated when they arrived at the clinic. On accessing patient charts, physicians were prompted to make an active choice to either: (a) accept or (b) cancel an order for influenza vaccine. | The study authors, including the Director of the Penn Medicine Nudge Unit. | An automated electronic health record prompt sent to physicians telling them to make an active choice. | Examination room (clinic) | The intervention was active from November 2010 to March 2013. | Not applicable | No modifications reported | Information not provided | Information not provided |
| Presseau et al (2018) | Going beyond knowledge: An educational intervention to improve prescribing for type 2 diabetes. | There is room for improvement in type 2 diabetes management in primary care.  This study assesses the effectiveness of a behavioral intervention to influence six clinician behavior: prescribing for blood pressure and glycemic control, providing physical activity and nutrition advice and providing updated diabetes education and foot examination.  Used Social Cognitive Theory, Health Action Process Approach, Dual Process Model and theory-based approaches to multiple goal pursuit. | Short videos using trained actors demonstrating patient-clinician interactions. | Intervention – Clinicians watched videos and then discussed the role of the six behaviors in their practice. They also discussed differences between current performance and intended levels, and identified barriers and enablers of providing physical activity and healthy eating advice. | A content expert (nurse or medical doctor) and a behavior change expert delivered the intervention. | Face-to-face interactions and short videos. | Primary Care | 90 min visit at each practice | Discussion at the practices were tailored as clinicians shared their own experiences, attitudes and beliefs about the six target behaviors. | No modifications reported | Fidelity of the intervention was assessed by coding transcribed audio recordings of all intervention sessions. These were analysed to judge whether intervention was delivered as intended. | High fidelity (further information not provided) Process evaluations planned as future research |
| Sacarny et al (2018) | Peer Comparisons: Quetiapine | Antipsychotic agents like quetiapine are overprescribed to older adults.  This study assesses the effectiveness of making people aware of other people’s prescribing practices to decrease quetiapine use.  The intervention used peer comparison messaging to nudge behavior, rooted in behavioral economics. | Letters.  Past prescription records. | Intervention – Prescribers at the 75^th^ percentile or higher for quetiapine use were sent an intervention letter. The intervention letter indicated that the prescriber’s quetiapine prescribing was high relative to their peers. The letter encouraged prescribers to review their prescribing patterns and to expect future communications from the Centres for Medicare and Medicaid Services. Two additional letters were sent with similar content. | The letters were sent by the Centres for Medicare and Medicaid Services | Mailed letters | Primary Care | The intervention was active from April 2015 to October 2015.  Each prescriber received three letters. | Information in letters was individually tailored such that prescribers would learn about their prescribing practices relative to their peers. | No modifications reported | Information not provided | Information not provided |
| Shakespeare et al (2019) | Education + medication chart adjustments: Analgesics | An audit identified that patients were often not given postoperative analgesic medication after caesarean section operation.  This study assesses the effectiveness of a two-part intervention to increase such use of analgesics.  Nudge theory adapted for better results. | Education materials for sessions.  Medication chart. | Intervention – The intervention was composed of two parts. First, education sessions were conducted with postoperative nursing staff to emphasize the importance of prescribing analgesics to patients after a caesarean section. Second, the medication charts were redesigned to include pre-printed medication orders. | A pain nurse, a pharmacist, and an anaesthetist led the education sessions. | Education sessions were conducted face-to-face, and the medication charts were altered. | Teaching Hospital | Information not available. | Individual conversations in educational programs involve personal feedback and advice. | No modifications reported | Information not provided | Information not provided |
| Yadav et al (2019) – Intervention 1 | An adapted stewardship intervention to reduce inappropriate prescribing: Antibiotics | To address the emerging threat from antibiotic resistance bacteria, prescribing must be more restricted.  This study assesses the effectiveness of a Center for Disease Control (CDC) adapted intervention to reduce antibiotic prescribing.  The intervention draws on economic theory, behavioral economics and decision science. | Education materials.  Physician champions.  Departmental feedback.  Brochures.  Campaign messages. | Intervention 1 – The adapted intervention incorporated strategies from the CDC’s Core Elements for Outpatient Antibiotic Stewardship: (1) provider education (presentations, guidelines and brochures), (2) patient education (posters and handouts), (3) provider commitments (personalized commitment posters in examination rooms), (4) a named champion, and (5) departmental feedback (monthly updates of prescribing practices). | A physician champion led the educational component. | Face-to-face interactions. | Hospital | The study interventions ran across three sites.  At two sites the interventions ran from July 2017 to February 2018.  At the third site the interventions ran from November 2017 to February 2018 | Local stakeholders at each site adapted materials provided by the CDC. | At two sites, the commitment posters were presented in the waiting rooms, rather than the examination rooms. | The fidelity of the intervention was assessed through risk analysis; deviations were recorded. Completion percentages were reported. | Planned interviews (100%)  Collection of public commitment signatures (99%)  Willingness to display public commitment (92.6%) |
| – Intervention 2 | An enhanced stewardship intervention to reduce inappropriate prescribing: Antibiotics | This intervention in the study assesses the effectiveness of an enhanced intervention to reduce antibiotic prescribing.  See ‘rationale’ and ‘theory’ in the above column. | In addition to above, emails were sent with peer comparison feedback. | Enhanced intervention – All elements of adapted intervention described above, and (6) peer comparison feedback (emails with personalized monthly performance rankings of each physician ranked as either a top decile performer or not a top performer). | See above column | Face-to-face interactions and emails | See above column | See above column | The information in the peer comparisons emails was tailored such that the provider would learn about their own performance compared to peers. | See above column | See above column | See above column |

Appendix 4. Quality assessments of the included studies based on SIGN’s checklists and notes.

| Study |  | Internal Validity | | | | | | | | | | Overall Rating |
| --- | --- | --- | --- | --- | --- | --- | --- | --- | --- | --- | --- | --- |
|  |  | The study addresses an appropriate and clearly focused question (Yes/ No/ Can't say) | The assignment of subjects to treatment groups is randomized (Yes/ No/ Can't say) | An adequate concealment method is used (Yes/ No/ Can't say) | The design keeps subjects and investigators ‘blind’ about treatment allocation (Yes/ No/ Can't say) | The treatment and control groups are similar at the start of the trial (Yes/ No/ Can't say) | The only difference between groups is the treatment under investigation (Yes/ No/ Can't say) | All relevant outcomes are measured in a standard, valid and reliable way (Yes/ No/ Can't say) | What percentage of the individuals or clusters recruited into each treatment arm of the study dropped out before the study was completed? | All the subjects are analyzed in the groups to which they were randomly allocated (often referred to as intention to treat analysis). (Yes/ No/ Can't say/ Not applicable N/A) | Where the study is carried out at more than one site, results are comparable for all sites (Yes/ No/ Can't say/ Not applicable) | How well was the study done to minimize bias? (High quality (++)/ Acceptable (+)/low quality (-)/ reject 0 |
| Lemiengre et al (2018) | Cluster randomized trial | Yes | Yes | Yes | Yes | Yes | Y | Yes | 19% | Yes | Not reported | ++ |
| Mafi et al (2018) | Non-randomized trial | Yes | Omitted | Omitted | Omitted | Yes | Not reported | Yes | Not reported | Yes | NA | - |
| Meeker et al (2014) | Individual randomized trial | Yes | Yes | Yes | Yes | Yes | Yes | Yes | 7% | Yes | Not reported | ++ |
| Patel et al (2018) | Non-randomized trial | Yes | Yes | Yes | Yes | Yes | Yes | Yes | 0% | Yes | NA | ++ |
| Patel et al (2017) | Cluster randomized trial | Yes | Omitted | Omitted | Omitted | Yes | Yes | Yes | Not reported | Yes | NA | + |
| Presseau et al (2018) | Cluster randomized trial | Yes | Yes | Yes | Yes | Yes | Yes | Yes | 0% | Yes | Not reported | ++ |
| Sacarney et al (2018) | Individual randomized trial | Yes | Yes | Yes | Yes | Yes | Yes | Yes | Not reported | Yes | Not reported | ++ |
| Yadav et al (2019) | Cluster randomized trial | Yes | Yes | Yes | Yes | Yes | Yes | Yes | 5% | Yes | Yes | ++ |
